# The Effectiveness of Ergonomic Training and Therapeutic Exercise in Chronic Neck Pain in Accountants in the Healthcare System: A Review

**DOI:** 10.7759/cureus.35762

**Published:** 2023-03-04

**Authors:** Charul Dandale, Priyanka A Telang, Pooja Kasatwar

**Affiliations:** 1 Department of Community Health Sciences, Ravi Nair Physiotherapy College, Datta Meghe Institute of Medical Sciences, Wardha, IND

**Keywords:** physiotherapy, therapeutic exercise, healthcare accountant workers, ergonomic training, chronic neck pain

## Abstract

Musculoskeletal illnesses or ailments that are linked to work-related risk factors are known as work-related musculoskeletal conditions. For this study, chronic neck pain is defined as the discomfort experienced between the C1 and C7 anatomic areas of the cervical spine as well as the adjacent muscles, excluding the shoulders. In the workplace, the term "ergonomics" refers to the interactions between workers and other workplace components. Clinically, deep cervical flexor training and retraining are used for treating neck pain and enhancing the capacity to maintain an upright posture. Ergonomic training and therapeutic exercises are significantly effective in reducing pain and disability and enhancing posture in the cervical region.

## Introduction and background

"Work style" is proposed as a mechanism by which ergonomic and psychosocial risk factors interact to affect the development, exacerbation, and/or maintenance of upper-limb pain and functional limitations [1]. Work-related musculoskeletal disorders (WRMSD) are injuries or disorders of musculoskeletal tissues that are associated with workplace-related risk factors [2]. WRMSD include cumulative trauma, repetitive strain, and overuse injuries [3].

Work-related musculoskeletal issues of the neck pose a significant problem for people who spend a significant amount of time using computers [4]. Musculoskeletal disorders (MSD) are the most common cause of long-term sick leaves and disability benefits or pensions in several industrial countries [5]. The annual prevalence of neck pain was estimated to range from 15% to 44% in community-based studies conducted worldwide [6]. Office workers have a higher prevalence of neck pain than the overall population. Globally, a one-year prevalence of neck pain among administrative workers has been reported to be between 15% and 43.3% [7,8].

As defined by the International Ergonomics Association (IEA), ergonomics is the scientific discipline concerned with understanding human interactions and other system elements [9]. Ergonomics is the science of making a worker's workplace fit, comfortable, safe, secure, and efficient to ensure that they enjoy providing productive outputs to the company they work for. The risk of numerous musculoskeletal problems is increased by today's compulsive use of gadgets and mobile devices, which should not be neglected. According to several researchers, the extended use of these devices causes improper postures and an increased risk of injury. Many researchers report that working 5.41 hours sitting at a desk and seven hours sleeping at night significantly impact physical and mental health. MSD are prevented and reduced with the help of ergonomic training methods (ETM), particularly in the neck and lumbar areas [10]. 

Therapeutic exercises, as another strategy, can address potential muscle imbalances that may contribute to the movement compensation that leads to neck pain. Clinically, deep cervical flexor training and retraining are used for treating neck pain and enhancing the capacity to maintain an upright posture. As therapeutic exercises have a significant impact, they are suggested for neck pain therapy in individuals with persistent neck discomfort, poor posture, and other health issues. O’Riordan et al. (2014) reported that resistance exercises and endurance training reduced pain and disability scores in office workers with chronic neck pain [11]. Although the therapeutic exercises used in the study aim to improve neck muscle control, strength, and postural control through strengthening and progressive resistance training, these techniques focus on neck and shoulder coordination and shoulder girdle power preservation. Maintaining excellent posture while undertaking exercises may be the key to the long-term retraining of therapeutic exercise efficacy.

## Review

Search strategy

The article reviews the impact of ergonomic training and therapeutic exercises on chronic neck pain in healthcare desk-job holders by evaluating and categorizing them with the help of the Neck Disability Index (NDI) based on certain inclusion criteria, including age range of 25-60 years and presence of chronic neck pain and poor posture, and exclusion criteria, including the presence of central nervous system diseases, muscle degeneration disorders, and rheumatic heart diseases. Databases of PubMed and Google Scholar were systematically searched till January 2021 using certain keywords, and Medical Subject Headings (MeSH) terms were used to find relevant articles. The articles were also screened using appropriate keywords related to ergonomic training, therapeutic exercises, and chronic neck pain. We extracted additional information from the references included in the articles; five studies were examined in the review article.

Method

Ergonomic Training

Ergonomic advice recommends ergonomic training while working on a desktop for the prevention of work-related upper-limb or neck musculoskeletal disorders. Repetitive tasks should be avoided, as they may increase tiredness. Tiredness may also be caused by an extraordinarily high or low workstation, making it difficult for the upper extremity to maintain its position for long periods, resulting in muscular fatigue. It is helpful to have one's forearms and hands in parallel and horizontal to the chair. While laying the fingers on the keyboard's middle row, the elbows should be vertically beneath the shoulder such that no angle is formed at the wrist joint. The feet should be flat on the floor. Every two hours, it is important to blink at regular intervals. For the best lighting, screen glare filters can be used. Lumbar pillows can be used if the chair is not made ergonomically. Sitting in an uncomfortable posture must be avoided, and changing positions frequently should be made into a habit. The screen should not be too close to the eyes, as this may cause vision-related problems, nor should it be too far to one side, as this puts an unequal amount of strain on the neck muscles. Both hands should be used symmetrically for work. Sitting on a chair with a footrest ensures that the pressure is relieved from the thigh and is equally distributed. To decrease neck and spinal strain at the office, a document holder can be placed on the table top. It should at be the same height and distance as the monitor. All the desktop accessories, such as phones, paper trays, and bottles, should be in an accessible position. Reaching and twisting should be avoided whenever possible. 

Ergonomic Training Along With Therapeutic Exercises

Therapeutic exercises, as another strategy, can address potential muscle imbalances that may contribute to the movement compensation that leads to neck pain. Clinically, deep cervical flexor training and retraining are used for treating neck pain and enhancing the capacity to maintain an upright posture. As therapeutic exercises have such a significant impact, they are suggested for neck pain therapy in individuals with persistent neck discomfort, poor posture, and other health issues. Although the therapeutic exercises used in the study aim to improve neck muscle control, strength, and postural control through strengthening and progressive resistance training, these techniques focus on neck and shoulder coordination and shoulder girdle power preservation. Maintaining excellent posture while exercising may be the key to the long-term retraining of therapeutic exercise efficacy. The ergonomic training and therapeutic exercises were most effective in the case of neck pain and impaired posture [12].

Discussion

The primary injury prevention program of office ergonomics training and therapeutic exercises positively affects workstation behaviors, lowers musculoskeletal risks, and ensures less stress in healthcare desk-job holders. The position of the monitor and the keyboard, the placement of the worker's elbows, forearms, upper arms, wrists, and shoulders while typing, and the position of the worker's lumbar support, thighs, knees, and feet while sitting see the most changes. In a six-month randomized trial, it was discovered that exercise and ergonomic changes, as well as exercise by itself, successfully reduced the degree of neck pain in desk-job holders [13]. In a case report by Fabrizio, the seat height of the patient's work chair was adjusted to enable the below-mentioned shoulder and elbow positions and the monitor viewing angle in combination with a relaxed leg and foot position. All other adjustments could be controlled from a platform provided by an adjustable chair [14]. Current evidence supports the performance of strengthening exercise therapies at the workplace with symptomatic office workers [15]. In a randomized control trial by Mahmud et al., workers benefitted from the change in workstation habits in terms of having excellent computing body postures and skills, which may minimize the risk of musculoskeletal illnesses in the future. Over time, workers in the training group reported fewer issues of the neck as well as the upper and lower back [16].

Individual, environmental, and psychosocial factors can be used to predict the likelihood of acquiring MSD [17]. Understanding the connection between computer use and MSD is crucial for preventing MSD from worsening. According to a study by d’Almeida et al. for a French company, managers and professionals are less likely to miss work due to upper-limb diseases than office and desk-job holders [18]. According to a population-based study conducted in Sweden, people who simultaneously reported neck-shoulder and lower-back discomfort were more likely to take both short-term and long-term sick leaves from work [19]. In this office ergonomics intervention research, employees who received a chair and office ergonomics training had less pain and discomfort during the workday compared to those who just received training or to a control group. When comparing the training-only group to the control group, there was no discernible difference in the symptom progression throughout the workday. This study had certain limitations as it could not consider the workplace standards or provide participants with more ergonomically sound workstations [20]. Pain is lessened, posture is improved, and disability is reduced when therapeutic exercises are combined with ergonomic training. It is more effective in reducing neck pain than other methods. In general, ergonomic training, in conjunction with therapeutic exercises, produces superlative outcomes in terms of increased strength, enhanced function, health-related quality of life, and decreased pain ratings. The review of the literature is given below in Table 1.

**Table 1:** Review of literature.

Sr. No	Title	Author, year	Study design	Main finding
1	Workplace-based Interventions for neck pain in office workers: systematic review and meta-analysis	Chen et al. (2018) [15]	Review article	Office workers who were experiencing neck pain were able to reduce it with the help of workplace strengthening exercises, and the effect was stronger when the exercises were specifically designed for the neck and shoulder.
2	A cluster-randomized trial of workplace ergonomics and neck-specific exercise versus ergonomics and health promotion for office workers to manage neck pain – a secondary outcome analysis	Johnston et al. (2021) [21]	Cluster- randomized trial	In order to reduce neck pain intensity in All Workers and those who experienced neck pain just after the intervention period, a combined exercise and ergonomic intervention performed better than combined health promotion and ergonomic intervention.
3	Effectiveness of workplace interventions in the prevention of upper extremity musculoskeletal disorders and symptoms: an update of the evidence	Van Eerd et al. (2016) [22]	Systemic Review Article	Workstation adjustment, electromyographic biofeedback training, and job stress management training alone therapies had a reasonable level of evidence that they had no impact on upper extremity musculoskeletal disorder and symptom outcomes. The degree of evidence for resistance training programs was quite strong.
4	Chronic neck pain and exercise interventions: frequency, intensity, time, and type principle	O’Riordan et al. (2014) [11]	Review Article	Increased strength, enhanced function, health-related quality of life, and decreased pain ratings all seem to be benefits of physical therapy that use a multimodal approach.
5	Ergonomic training reduces musculoskeletal disorders among office workers: results from the 6-month follow-up	Mahmud et al. (2011) [23]	Randomized control trial	At the follow-up time point, consistent decreases were seen for all musculoskeletal conditions. Office workers who received ergonomic training had better workstation habits and experienced fewer musculoskeletal ailments.

## Conclusions

The current study showed that it is helpful for medical accountants to receive ergonomic training alongside therapeutic exercises in potentially improving their posture and reducing neck pain. Stretching and isometrics are included therapeutic exercises. Ergonomic training, combined with therapeutic exercises, decreases pain, improves posture, and reduces impairment. It has a high success rate in relieving discomfort in the cervical area. In terms of enhanced strength, improved function, health-related quality of life, and lower pain ratings, ergonomic training, combined with therapeutic exercises, offers excellent results. The effectiveness of therapeutic exercises and ergonomic training programs can benefit the medical accountant working population.
